# The Nervous System and Its Constituent Neurones

**Published:** 1900-01

**Authors:** 


The Nervous System and its Constituent Neurones. Designed for the use of practitioners of medicine, and of students. By Lewellys F Barker, M. D., Associate Professor of Anatomy in the Johns Hopkins University, and Assistant Resident Pathologist to the Johns Hopkins Hospital. With two colored plates and 676 illustrations in the text. Octavo, pp. 1122. New York: D. Appleton & Co. 1899.

This work represents the results of long and careful study and scientific research in the anatomy, histology and physiology of the nervous system in man. It is, moreover, a subject which has since the beginning of scientific records, occupied the minds of thoughtful physicians and observers the world over. Within the last decade a complete revolution in our ideas concerning the elements of the nervous organs has taken place and as a result new methods of investigation have been discovered, new avenues of research have been opened up, problems hitherto thought unsolvable have been solved and the key to the whole intricate subject, the neuron theory, been promulgated and accepted by the great majority of thinking men, active in the domain of neuro-anatomy and neuro-histology.

The author began his work some two years ago in a series of articles running in the *New York Medical Journal*, in which an attempt was made to present, in as simple and concise a manner as possible, the main facts concerning the newer investigations into some phases of the anatomy and physiology of the nervous system. Those who fortunately read those articles will be pleased to know that they form hardly more than an introduction to the present volume and the body of the book dealing with the groups of neurones, whose axones constitute the principal known tracts in the nervous system—centripetal, centrifugal and associative—is now published for the first time.

The neurone is defined to be the nervous cell in its entirety; its central mass branches and connections by which the vital matter of the nerve cell is connected with the vital matter of all other vital cells, the branches being designated dendrites, and connecting structures axis-cylinders or axones. The purpose of the author in this work is to show the actuality of such structures, and that the whole nervous system consists of the grouping of neurones.

The opening chapter is devoted to the history of the development of the neurone concept in which appear the labors of the early neuroanatomists Schwann, Golgi, Deiters, Waldeyer, Forel, His and the brilliant later researches of Ramon y Cajal with the Golgi method. It is a curious fact that the method to which the whole modern fabric of the neurone is due was unused and neglected for more than twelve years after its discovery by Golgi in 1873. Since then it has produced results in the hands of expert observers, most important and path-making in the science of neurology.

Section II. takes up the external morphology of the neurones, describing minutely and carefully the external form of the cell body and of the dendrites; the external form of the axis cylinder processes or axones and the collaterals, side fibriles, and interneuronal substances. The methods of Weigert, Nissl, Apáthy and Held have done much to unravel the mysteries of the finer anatomy of the neurone components and their importance is given due consideration by the author in these pages.

Section III. is devoted to the internal morphology of neurones. In this department perhaps the greatest improvements and discoveries have been made and to those who desire to be abreast of the times this chapter will be interesting reading. The status of our knowledge about the internal structure of the protoplasm may be summed up as follows: A neurone is made up like other cells of nucleus, and protoplasm. In the latter a centrosome and a so-called attraction sphere are present. The protoplasmic portion of the cell can be divided into a peripheral exoplasmic and a central endoplasmic portion. In neurones as in muscle cells there is a tendency to a fibrillory structure. In both exoplasm and endoplasm there can be made out a more or less homogeneous ground substance in which are deposited larger and smaller masses of a granular nature. The ground substance corresponds to the "unstainable substance" of Nissl, and the masses of granules to the stainable substance of Nissl and the pigment.

Then follow chapters on the histogenetic relations of the neurone as a unit in physiological and pathological processes and the grouping and chaining together of neurones in a complex nervous system, like that of man and higher animals.

Too much cannot be said in commendation of this work. It will be greeted with pleasure by everyone who desires to understand the phenomena of life and the wonders of the nervous system; that structure of the living organism that connects all the living parts of the body and makes it a consistent and harmonious whole.

The illustrations, very profuse and elegant, show such a connection and grouping and make clear how the living cells of every organ take part in the operation of life throughout the organism.

On the whole the work is worthy of the highest praise and commendation, well written carefully worded, free from misspelled proper names and is surely a credit and honor to American neurology.

The publishers have done their work so well that any praises would be superfluous. W. C. K.

